# Nano-encapsulated ferulic acid in sesame protein isolate alleviates acrylamide-induced liver toxicity and genotoxicity in rats via oxidative stress and DNA damage modulation

**DOI:** 10.1186/s40360-025-00946-8

**Published:** 2025-06-13

**Authors:** Hend A. Essa, Elham Ali, Fatma El Zahraa Abd EL Hakam, Engy M. Akl

**Affiliations:** 1 Nutrition and Food Sciences Department, Food Industries and Nutrition Research Institute, National Research Centre, 33 El Bohouth St, Dokki, Giza 12622 Egypt; 2 Molecular Biology, Zoology and Entomology Department, Faculty of Science (for Girls), Al-Azhar University, Nasr City, 11754 Cairo, Egypt; 3 Pharmacology department, Faculty of Medicine (for Girls), Al-Azhar University, Nasr City, Cairo, 11754 Egypt; 4 Fats and Oils Department, Food Industries and Nutrition Research Institute, National Research Centre, 33 El Bohouth St, Dokki, Giza 12622 Egypt

**Keywords:** Ferulic acid, Genotoxicity, Liver damage, Oxidative stress, Acrylamide, Sesame protein isolate, Encapsulation

## Abstract

**Background:**

Acrylamide (ACR) induces hepatotoxicity and genotoxicity through oxidative stress and inflammatory processes.

**Aims:**

This study explores the potential of ferulic acid encapsulated in sesame protein isolate (SPI) and its nanoform as a non-toxic, effective therapy for ACR-induced oxidative liver injury in rats.

**Methods:**

SPI was prepared from defatted sesame flour. SPI exposed to ultrasonic waves to obtain nano SPI, and then ferulic acid was added to form capsules. Fourier transforms infrared spectra, scaning electron microscope, and polarizing optical microscope were used in investigating functional groups and surface morphology of both encapsulations respectively. Rats were divided into four groups, each consisting of six animals: normal control, ACR-treated (20 mg/kg/day), sesame protein encapsulated ferulic acid-treated, and sesame protein nano-encapsulated ferulic acid-treated groups. Both encapsulated forms were administered daily in the diet alongside ACR for two weeks. Liver function indices, oxidative stress biomarkers, DNA fragmentation, comet assay, and histopathological and immunohistochemical examinations were performed.

**Results:**

The encapsulation efficiency of the nano-encapsulated form was higher than that of the other forms. Both encapsulated forms significantly improved liver function, elevated levels of GSH, GPx, SOD, and CAT were observed, along with decreased concentrations of MDA, interleukin-6, and tumor necrosis factor-α. The treatments also provided protection against DNA damage and genotoxicity, alleviated histological damage, and reduced liver toxicity and genotoxicity.

**Conclusion:**

Both encapsulated forms, especially the nanoform, significantly mitigated liver toxicity. These findings underscore their potential as effective natural therapies for liver damage caused by ACR, and supporting liver health.

**Supplementary Information:**

The online version contains supplementary material available at 10.1186/s40360-025-00946-8.

## Introduction

Acrylamide (ACR), with the molecular formula C3H5NO, is a water-soluble compound commonly used in polymer production [1]. It has been demonstrated that acrylamide forms when carbohydrate-rich foods are subjected to high-temperature cooking (above 120 °C). This process occurs via a Maillard reaction between reducing sugars, such as glucose, and amino acids like asparagine. This process occurs during cooking methods like roasting, grilling, baking, and frying [2]. Consequently, individuals who frequently consume foods cooked at elevated temperatures may ingest approximately 0.5 mg/kg/day of ACR [3].

ACR is also extensively applied across various industries, including cosmetics, paper manufacturing, textiles, wastewater treatment, and printing, and it plays a role in polymers and copolymers production [4]. However, ACR exposure has been linked to numerous toxic effects, particularly hepatotoxicity, and genotoxicity [5].

The primary mechanism underlying ACR’s toxicity is oxidative stress. ACR is metabolized by pathways involving glutathione or cytochrome P450, resulting in the production of glycidamide, a metabolite with higher toxicity than ACR itself [6]. It has been indicated that exogenous antioxidants can hinder glycidamide formation, thus mitigating oxidative damage [7]. Elevated doses of ACR have been shown to disrupt the oxidative balance and enzyme function, increasing oxidative stress and leading to organelle damage, cellular metabolic disruption, DNA fragmentation, and cell death [8].

Research indicates that phenolic compounds may offer protection against oxidative damage and ACR’s associated toxic effects [9, 10]. Ferulic acid (FA), a phytochemical with the molecular structure C10H10O4, is naturally present in the cell walls of various plants, including grains (such as rice, wheat, and oats), nuts, citrus fruits, coffee, tea, and peanuts. FA has been documented to provide multiple health benefits, including antioxidant, anti-apoptotic, hepatoprotective, anti-inflammatory, and anti-carcinogenic properties [11].

The broad range of preventive effects associated with FA is attributed to its potent antioxidative properties. In animal models, it has demonstrated the ability to prevent methotrexate-induced liver toxicity and reduce oxidative stress, inflammation, and apoptosis by enhancing Nrf2/HO-1 signaling pathways [12].

However, FA ‘s bioavailability and stability are limited. Therefore, encapsulating FA within nanocarriers can enhance its delivery and efficacy. Sesame protein is a promising natural biopolymer for encapsulation due to its high binding affinity, biodegradability, and antioxidant properties [13, 14].

Sesame (Sesamum indicum L.), An essential oil seed crop, is a member of the Pedaliaceae family with a high protein content (30–60%), about 50% oil, and a wide variety of phytochemicals, including phenolics, carotenoids, tocopherols, and phytosterols [15–17]. Sesame seeds exhibited a wide range of biological properties, including antidiabetic, anticancer, cardioprotective, and antioxidant properties [18]. Sesame protein isolate (SPI) is amphiphilic, enabling the formation of nanoparticles through hydrophobic interactions, enhancing the stability and controlled release of encapsulated bio-actives [19]. SPI was prepared from defatted sesame flour and utilized for human and animal nutrition due to its protein quality [20]. Ultrasound (US) technology has been employed to modify protein structures, reducing particle size and altering functional properties. These modifications have been shown to enhance the encapsulation efficiency and bioactive stability of FA, as demonstrated with soy and peanut proteins [21–23]. Encapsulation technology allows bioactive molecules, such as FA, to be enclosed within a protective matrix, improving their stability and controlled release in physiological environments [24].

Therefore, this study aimed to evaluate the therapeutic effects of FA encapsulated in SPI and its nanoform against ACR-induced liver toxicity and genotoxicity in rats. By leveraging sesame protein as a natural encapsulating agent and employing nanotechnology, this research introduces an innovative approach to enhance the stability, and bioavailability of FA, providing a novel and effective strategy to mitigate oxidative stress, genotoxicity, and liver damage caused by ACR.

## Materials and methods

### Materials

#### Chemicals

Sesame seed, Sodium alginate (SA), 1,1-Diphenyl-2-picryl-hydrazyl (DPPH•), ABTS 2,2′-azino-bis-(3- ethylbenzothiazoline-6-sulphonic acid), ferulic acid, all chemicals and reagents used in the study were of analytical grade and applied without further purification Acrylamide (Sigma-Aldrich, St. Louis, MO, USA).

### Methods

#### Preparation of sesame protein isolate (SPI)

Sesame seed meal, which was already hydraulic pressed, was subjected to defatting using a soxhlet apparatus and n-hexane as a defatting solvent. The defatted meal was spread to dry and then ground in a coffee mill to obtain a finely divided material suitable for further extraction. Generally, the Sesame seed meal was dissolved in ten volumes of distilled water, and SPI was prepared by the iso precipitation method as described by Mohamed et al. [25]. The dried SPI was saved in the refrigerator until used.

#### Preparation of SPI capsules and nano SPI capsules

SPI (150 g) was hydrated to 10% w/v in dist. water overnight at room temperature (21˚C). Sodium alginate 10% w/v dissolved in dist. water by gentle magnetic stirring at 60 ℃ for 20 min. for complete hydration. SPI solution was mixed with sodium alginate in a ratio of 9:1 by gentle stirring for 1 h. at pH 7. FA was then added drop by drop to the wall material 1%w/w by gentle stirring for 20 min.

SPI nanoparticles were prepared as described by El-Kholy et al. [26] with some modifications. SPI (150 g) was hydrated to 10% w/v in dist. water overnight at room temperature (21˚C). Nano protein solution was formed using high-speed homogenizer T18 basic (IKA, Wilmington, USA), operating at a speed of 20,000 rpm for 5 min, then ultrasonicated at 720 W power, for 20 min at 25 °C and with 50% pulse using an ultrasonic with a titanium probe (vibra cell, USA). The solution temperature was controlled to be ≤ 35 ◦C in an ice-water bath. Then it followed the same steps in preparation of SPI capsules.

Both capsules were freeze-dried (by CHRIST Alpha 1–4 LD plus, Germany) at-42 °C under a pressure of 10 Pa for 48 h and stored at -4 °C. After freeze-drying, the samples were stored at 4 ℃ until future use.

#### Characterizations of prepared SPI

##### Determination of amino acid profile of Sesame protein isolate using HPLC

The amino acid profile was determined as described by Younos & Akl [27]. It was determined using the HPLC-Pico-Tag method, a commercially developed technique by Waters Associates for integrated amino-acid analysis.

##### Particle size distribution of nano SPI

The colloidal stability properties, including the demonstration of hydrodynamic diameter (HD) based on dynamic light scattering techniques, have been investigated using the Malvern Zeta Sizer Nano ZS Nano instrument with He/Ne laser (i.e., λ = 633 nm) at 173 °C collecting backscatter optics. The colloidal properties of samples were examined by adding about 2 ml of the sample and dispersing with 1 ml dist. H_2_O for 10 min under ultrasonication before measurements.

##### Fourier transforms infrared spectra

Functional groups were detected in the range of 400–4000 cm − 1 using (Shimadzu 8400 S) FTIR Spectrophotometer.

##### **Scan electron microscope (SEM)**

**SEM** studying the surface morphology (FEI IN SPECTS Company, Philips, Poland) environmental scanning without coating with a JEOL JEM-2100 electron microscope at 100k x magnification and an acceleration voltage of 120 kV.

##### Polarizing optical microscope (POM)

**The images were taken by** Leica DM750P (Leica microsystems. Switzerland) **(**Optical magnification 400 times, digital magnification 4 times).

##### **Encapsulation efficiency** (**EE**)

**EE** was determined according to the method established by Siles-S´anchez et al. [28] 45 mg of encapsulated capsules were suspended in 5 ml of water and agitated for 10 min. The supernatant containing non-encapsulated compounds (500 µl) was centrifuged at 3,000xg for 15 min. EE% was calculated using the following equations: EE(%) = 100 - (sum of the supernatant phenolic compounds /sum of the phenolic compounds in extract)×100.

##### Evaluation of antioxidant activity by the following methods

###### Radical DPPH scavenging activity

Free radical scavenging capacity was determined using the stable 1,1-Diphenyl-2-picryl-hydrazyl (DPPH•). The approach given by De Ancos et al. [29] was used to calculate DPPH radical scavenging. The decrease of the DPPH radical was detected at 517 nm. The assay was performed in triplicate. The results were represented as percent inhibition of the DPPH using the following equation:

$$\:Inhibition\:\left(\%\right)\hspace{0.17em}=\hspace{0.17em}100\:\times\:\:(A_{control}-A_{sample})/A_{control}$$

Where: A control: the absorbance of the methanolic DPPH solution, A sample: the absorbance of the extract.

###### ABTS radical scavenging capacity

Free radical scavenging activity was determined by ABTS radical cation decolorization assay according to Re et al. [30]. ABTS·+ radical cation was produced by the reaction between 7 mM ABTS in water and 2.45 mM potassium persulfate (1:1), stored in the dark at room temperature for 12–16 h. before use. ABTS·+ solution was then diluted with methanol to obtain an absorbance of 0.700 at 734 nm. After the addition of 5 µl of sample to 3.995 ml of diluted ABTS·+ solution, the absorbance at 734 nm was measured 30 min after the initial mixing. The ABTS scavenging effect was measured using the following formula:

$$\:Inhibition\:\left(\%\right)\hspace{0.17em}=\hspace{0.17em}100\:\times\:\:(A_{control}-A_{sample})/A_{control}$$

Where: A control: the absorbance of ABTS solution, A ample: the absorbance of the extract.

#### Animals

In this study, male Sprague Dawley rats aged 4 to 6 weeks and weighing 200–240 g were sourced from the animal facility of the National Nutrition Institute, Egypt. The animals were allowed a one-week acclimatization period, during which they were housed under standardized laboratory conditions. Each rat was individually placed in a polypropylene cage, with the environment maintained at a temperature of 25 °C, a relative humidity of 55%, and a 12-hour light/dark cycle. The rats were provided with unrestricted access to a standard laboratory diet and water throughout the experiment.

#### Diet composition

A balanced diet was formulated in accordance with the AIN-93 guidelines outlined by Reeves et al. [31]. The composition included 12% protein derived from casein, 10% corn oil, 10% sucrose, 58.5% corn starch, 5% cellulose, 3.5% salt mixture, and 1% vitamin mixture. Both the salt and vitamin mixtures were prepared based on the AIN-93 formulation, as described by Reeves et al. [31].

#### Ethical statement

This study was approved by the National Nutrition Institute Ethics Committee (ethical approval number: [IN000147]). Furthermore, the research protocol strictly adhered to the guidelines for the care and use of laboratory animals as established by the National Institutes of Health, detailed by Garber et al. [32].

#### Experimental design

Twenty-four male Sprague-Dawley rats were divided into four groups (6 rats per group) as follows:

- Group 1: Normal control group. Rats were orally administered normal saline (0.5 mL/rat) and fed a standard balanced diet daily.
- Group 2: ACR group. Rats were orally administered ACR (20 mg/kg/day) daily for 2 weeks [33]. This has been reported to induce total liver toxicity [34].
- Group 3: ACR + Sesame protein encapsulated FA. Rats were orally administered 20 mg/kg ACR daily and fed a balanced diet supplemented with 0.1% FA Zhang et al. [35] and 5% sesame protein in encapsulated form.
- Group 4: ACR + Sesame protein Nano-encapsulated FA. Rats were orally administered 20 mg/kg ACR daily and fed a balanced diet supplemented with 0.1% FA Zhang et al. [35] and 5% sesame protein in nano-encapsulated form.

All rats in the study were consistently provided with the assigned diet and water *ad libitum*. Throughout the study, body weight and food consumption were monitored on a weekly basis to assess any changes over time.

##### Growth-related parameters

At the conclusion of the 2-week study, body weight gain and relative liver weight were evaluated using the methodology described by Chapman et al. [36].

#### Blood sampling and preparation of liver homogenate

The animals were euthanized 24 h after the 14th dose of ACR administration. Thiopental sodium (50 mg/kg) was used to anesthetize the rats prior to euthanasia. Blood samples were collected via decapitation while the animals were under deep anesthesia. Serum was separated by centrifugation at 3000 rpm for 15 min at 4 °C using a Laborezentrifugen 2k15 centrifuge (Sigma, Germany) and stored at -20 °C for subsequent biochemical analyses. Liver tissues were immediately excised, rinsed with ice-cold saline, blotted dry with filter paper, and weighed. The liver was divided into three portions: one portion was snap-frozen in liquid nitrogen and stored at -80 °C for genotoxicity assessments, the second was preserved in 10% (v/v) neutral buffered formalin for histopathological evaluation, and the third, comprising one gram of tissue, was homogenized in ice-cold phosphate-buffered saline (pH 7.4) to prepare a 20% w/v homogenate using an MPW-120 tissue homogenizer (BitLab Medical Instruments, Poland). The homogenate was centrifuged at 4000 rpm for 10 min at 4 °C using a Laboratory Centrifuge 2 K15 (Sigma, Germany), as described by Essa et al. [37]. The resulting supernatant was collected, stored at -80 °C, and used for measuring oxidative stress markers and inflammatory parameters.

#### Liver injury assessment

The evaluation of hepatic function biomarkers and protein fractions included the following: Serum levels of alanine aminotransferase (ALT) and aspartate aminotransferase (AST) were measured using the Reitman and Frankel method 1975 [38]. Alkaline phosphatase (ALP) levels were determined according to the procedure by Bessey [39], while gamma-glutamyl transferase (γ-GT) was assessed as outlined by Szasz [40]. Total protein and albumin levels were quantified using the methods described by Rheinhold [41] and Doumas [42], respectively. Additionally, total and direct bilirubin were measured according to the methodology of Balistreri and Shaw [43]. All assays were performed using commercial kits from Salucea Co., Netherlands.

#### Assessment of oxidative stress markers

Hepatic malondialdehyde (MDA), superoxide dismutase (SOD), catalase (CAT), glutathione peroxidase (GPx), reduced glutathione (GSH), and nitric oxide (NO) levels were evaluated spectrophotometrically using the methods outlined by Nair and Turner [44]; Sun [45]; Luck [46]; Rotruck et al. [47]; Jollow [48]; and Montgomery and Dymock [49], respectively. Biochemical analyses were carried out in accordance with the manufacturer’s instructions. The optical density of all measured parameters was determined using a Shimadzu UV-2401 PC spectrophotometer (Australia). Spectrophotometric analyses were conducted in accordance with the guidelines provided with the bio diagnostic kits.

#### Determination of TNFα and IL-6 in liver tissue

Levels of TNF-α and IL-6 were measured in liver homogenate using the rat TNF-α ELISA kit (Sunlong Biotech Co., Catalog no. SL0722Ra, China) and the IL-6 ELISA kit (Sunlong Biotech Co., Catalog no. SL0411Ra, China), following the sandwich-ELISA method and spectrophotometric analysis as per the instructions provided with the kits.

### Histopathological studies

#### A. Hematoxylin and Eosin (H/E)

Liver specimens were fixed in a 10% formalin saline solution. After dehydration in different grades of ethyl alcohol (100%, 5 min; 95%, 2 min; 80%, 2 min; 70%, 2 min), cleaning in xylol, impregnation, and proper fixing, we embedded the specimens in paraffin wax (5 mm thick pieces). Then, using a rotatory microtome (LEICA RM 2125 UK), specimens were cut with a 5 μm thickness and placed on glass slides. Staining with hematoxylin and eosin (H/E) solutions (G1120, Solarbio, China) for 30 min at 55 ℃ was used to explore the lung’s general histological structure under a light microscope [50].

For Oil Red O Staining, the samples were rinsed with PBS and fixed in 10% buffered formalin, then stained with Oil Red O (0.5 g in 100 mL of isopropanol) for 60 min. After discarding the staining solution, isopropanol was added to the samples to elute the retained dyes [51]. After mounting, the sections were visualized and photographed using an optical microscope with a camera (Olympus, Tokyo, Japan) at 100× or 400× magnification. Slides were examined under a light microscope (Primo star, ZEISS, China). The photos were taken using (Axiocam Erc 5s, ZEISS, China) camera, at the pathology department, faculty of Medicine for Girls, Al Azhar University.

#### B. Immunohistochemical study

**Apoptosis and Caspase-3 assessment** Regarding immunohistochemistry stain, liver paraffin slices were deparaffinized and rehydrated on positively charged glass slides. After 30 min of incubation in 0.3% hydrogen peroxide in 100% methanol, endogenous peroxidase was rendered inactive. At room temperature, sections were incubated for 30 min in 5% skim milk. Microwave (700 W) treatment in 10 mM citrate buffer (pH 7.4) for 15 min was used to retrieve the antigen. After that, sections were incubated with anti-rat caspase-3 for an entire night at 4ºC. Sections were treated in secondary antibodies for 30 min at room temperature following PBS washing. After adding 3-diaminobenzidine for two to four minutes, washing in distilled water, then counter-staining with Mayer’s haematoxylin for one minute at room temperature, a brown hue forms [52, 53].

#### DNA fragmentation assay

DNA extraction was done from liver tissue (200 mg) by using the Zymoresearch Quik-gDNA^™^ MiniPrep kit (CAT. NO. D3024) following the manufacturer’s instructions. DNA extraction was quantified as optical density (OD) using a spectrophotometer at 260 nm. DNA fragmentation was detected as 20 µg of DNA sample was loaded on 2% agarose gel electrophoresis (Ultra-pure agarose, electrophoresis grade) with Ethidium Bromide (0.5 µg/ml). DNA visualization and documentation were done under a UV transilluminator using a DNA ladder,100 bp (Jena Bioscience, Germany), and gel documentation system (Biodoc. Analyser (Biometra).

#### Alkaline comet assay (single-cell gel electrophoresis)

According to the manufacturer’s instructions, a comet assay test was conducted to detect the DNA strand breaks using COMET ASSAY™ PROTOCOLS (Ams Biotechnology, Europe, ltd), CAT. # 4250-050-K (Comet Assay™ Kit). It is a sensitive technique that enables analysis at the single-cell level.

### Statistical analysis

Statistical analyses were conducted using SPSS version 25. The data obtained from the animal experiments were expressed as mean ± standard error (SE) and analyzed using a one-way analysis of variance (ANOVA) followed by Duncan’s multiple range test. Statistical significance was established at a threshold of *P* ≤ 0.05.

## Results

### Characterizations of SPI

**Fig. 1:**
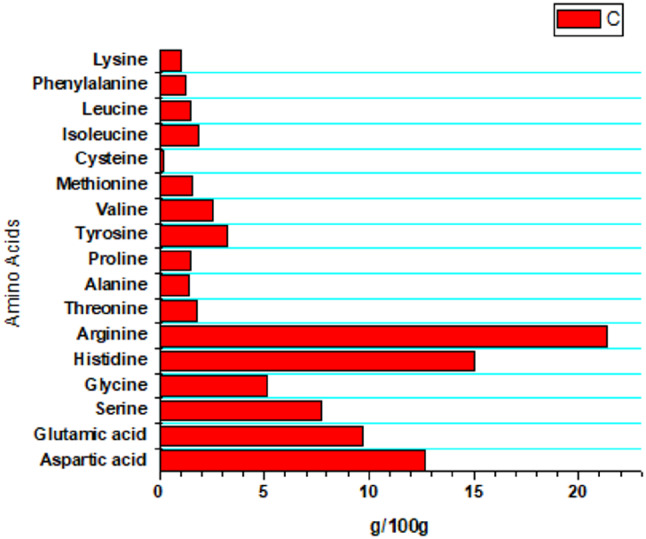
Amino acid profile of SPI expressed as gram for each 100 gram

**Fig. 2:**
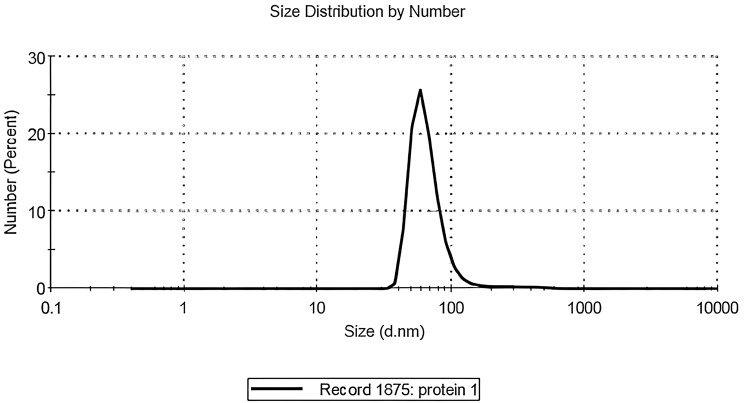
The particle size of the prepared nano SPI

The amino acid profile of SPI showed that Arginine was the most abundant amino acid, followed by Histidine, Aspartic acid, and Glutamic acid, as shown in Fig. 1. The size of soluble prepared nano SPI is represented in Fig. 2, showing a peak with a percentage of 100% at 71.08 ± 47.66 nm.

### Fourier transforms infrared (FTIR) spectroscopy

**Fig. 3:**
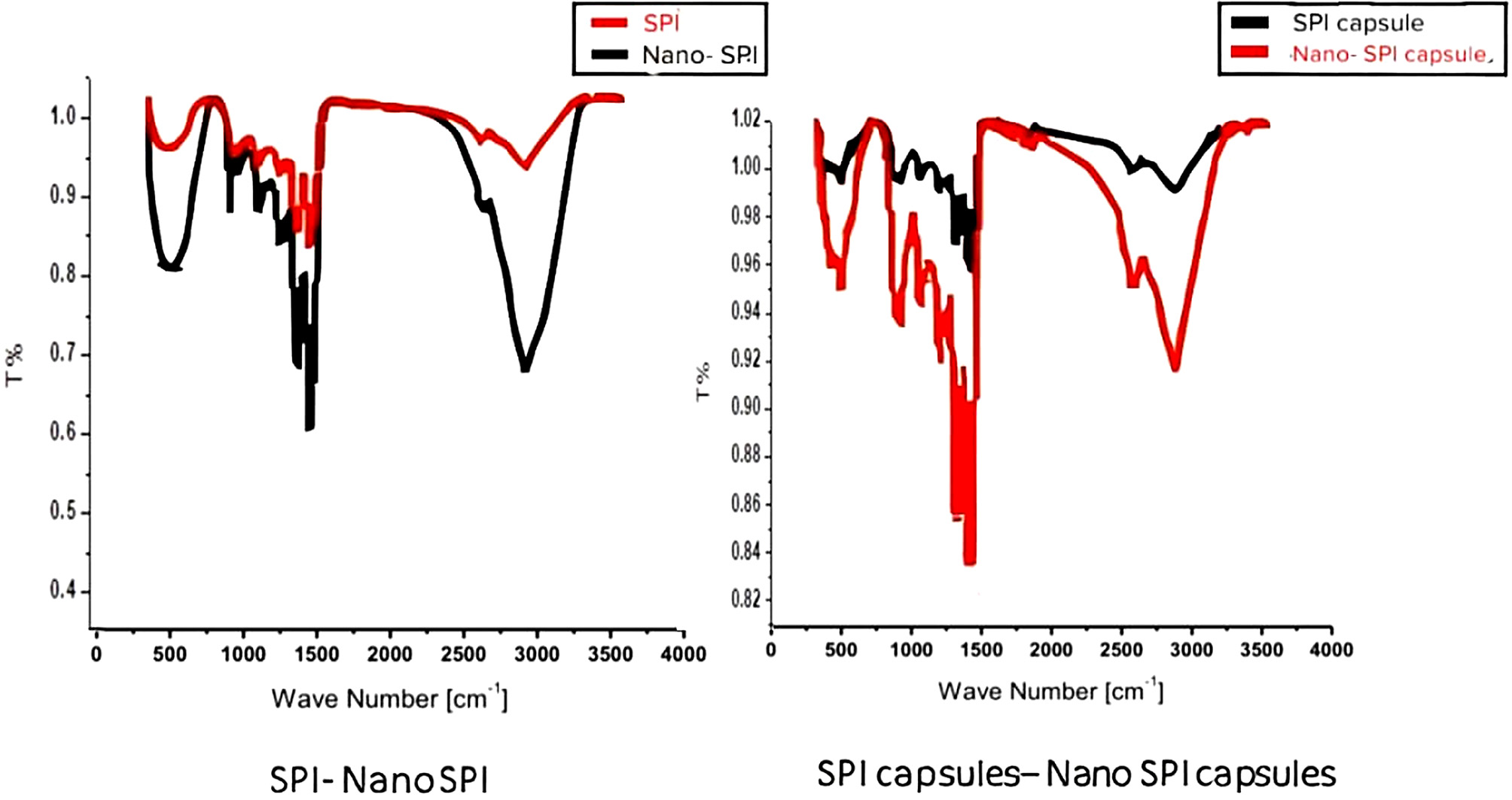
Fourier transform infrared spectrophotometer. A(SPI and nano SPI, B(SPI encapsulated and nano SPI encapsulated

The most displayed functional groups detected by FTIR spectra of SPI, and nano SPI, SPI encapsulated, and nano SPI encapsulated were represented in Fig. 3. The spectrum of SPI consists of several bands located at 3273, 2962 cm^− 1^, 1669, 1632 and 1537 cm^− 1^. The spectrum of the nano form of SPI showed a similar pattern of bands as those of SPI. However, the bands’ intensity was changed. FTIR spectrum of both encapsulated forms displays some difference in frequency and intensity. It shows a broad band at 3279–3732, 2924, 1633 and 1529 cm^− 1^ which are assigned to the O-H, C-H, C = O, C-N and N-H groups of peptide groups. In nano encapsulated form, the bands’ intensity was changed, but it still shows the same bands at 3276–3707, 2967, 1632, 1531, and 699–407 cm-1 as detected in SPI encapsulated.

### The morphological structure of each encapsulated SPI investigated by polarized optical microscope (POM) and scan electron microscope (SEM)

**Fig. 4:**
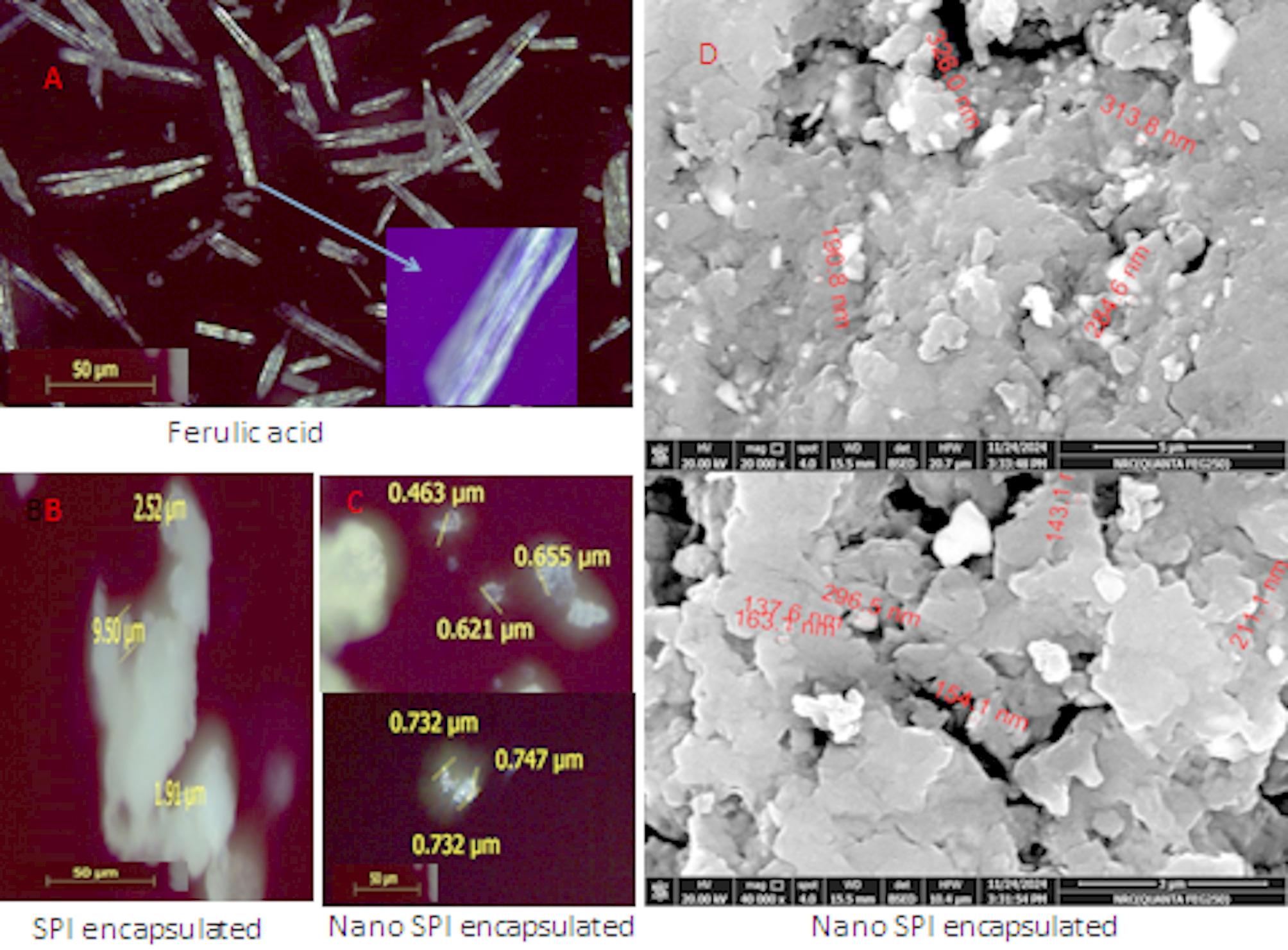
Shows POM image of **A**) ferulic acid, **B**) SPI encapsulated, **C**) nano SPI encapsulated. Optical magnification 400 times, digital magnification 4 times. **D**) SEM image of nano SPI encapsulated

POM images revealed that FA appeared as needle-like crystals as shown in Fig. 4A. Additionally, both types of protein, whether in micro or nano size exhibited a uniform spherical shape (Fig. 4B and C). SPI particles were found to range from 1.91 ± 0.2 μm to 9.50 ± 0.3 μm (Fig. 4B), while SPI nanoparticles ranged from 439 ± 4 nm to 747 ± 8 nm in the dry form as shown in Fig. 2C. The SEM image in Fig. 4D shows that FA appears as lightening crystals surrounded by nano SPI. This also confirms the formation of nano-protein which ranged from approximately 100 ± 5 nm:400 ± 10 nm.

### Encapsulation efficiency (EE) and antioxidant activity of each SPI encapsulated

Encapsulation efficiency (EE) of nano SPI encapsulated 85.35 ± 1.2 is higher than that of SPI encapsulated (67.41 ± 0.8) which illustrates the importance of nano form particles. The Antioxidant activity of the soluble capsules also showed that the percent scavenging activity of DPPH and ABTS percentage of SPI encapsulated is higher in SPI encapsulated (90.22 ± 0.5, 77.36 ± 0.7) than nano SPI encapsulated (77.65 ± 0.4, 68.88 ± 0.3), respectively as shown in Table 1.

**Table 1:** Encapsulation efficiency (EE) and Antioxidant activity of both capsules

	SPI encapsulated FA	Nano SPI encapsulated FA
**Encapsulation efficiency %**	67.41 ± 0.8^b^	85.35 ± 1.2^a^
	**Antioxidant activity**	
**DPPH %**	90.22 ± 0.5^a^	77.65 ± 0.4^b^
**ABTS%**	77.36 ± 0.7^a^	68.88 ± 0.3^b^

Results are mean values of three replicates ± standard deviation. Different letter(s) in the same row indicates significant differences at *p* < 0.05.

### Effect of SPI encapsulated FA and nano SPI encapsulated FA on nutritional parameters, liver function, hepatic oxidative stress, and inflammatory markers

**Table 2:** Nutritional parameters, liver weight, and liver index of different experimental groups

Groups	Normal group	ACR group	SPI encapsulated FA	Nano SPI encapsulated FA
Parameters				
Initial body weight (g)	206.2 ± 4.52^a^	206.3 ± 3.84 ^a^	206.2 ± 4.16 ^a^	206.2 ± 3.97 ^a^
Final body weight (g)	248.38 ± 6.11 ^a^	226.97 ± 5.22^b^	237.93 ± 4.32^c^	245.87 ± 4.41^d^
Body weight gain (g)	42.18 ± 3.20^a^	20.67 ± 2.11^b^	31.73 ± 2.85 ^c^	39.67 ± 3.05 ^a^
Total food intake (g)	281 ± 18.22 ^a^	216 ± 15.41 ^b^	238.2 ± 16.05 ^c^	259.4 ± 16.92 ^d^
Feed efficiency ratio	0.15 ± 0.015 ^a^	0.096 ± 0.01 ^b^	0.13 ± 0.01 ^c^	0.15 ± 0.01 ^a^
Relative liver weight (%)	1.75 ± 0.03 ^a^	2.53 ± 0.05 ^b^	2.34 ± 0.04 ^c^	2.11 ± 0.03 ^d^

In each row, the same letters mean non-significant difference while different letters mean significant difference at *p* < 0.05. The data are expressed as mean values ± standard error for 6 rats in each group. Feed efficiency = (body weight gain/total food intake)

As shown in Table 2, the ACR group showed significant (*p* < 0.05) reductions in final body weight, body weight gain, and total food intake by 8.6%, 51%, and 23.1%, respectively, compared to the normal group, alongside a 1.45-fold increase in relative liver weight, indicating hepatic stress. Treatment with **SPI** encapsulated FAsignificantly improved these parameters, with final body weight, body weight gain, and food intake increasing by 4.8%, 53.5%, and 10.3%, respectively, compared to the ACR group, while relative liver weight decreased by 7.5%. **Nano SPI** encapsulated FA demonstrated superior ameliorative effects, restoring final body weight, body weight gain, and food intake to near-normal levels (8.3%, 91.9%, and 20.1% improvement, respectively, vs. acrylamide group) and reducing relative liver weight by 16.6%.

**Table 3:** Liver function parameters of different experimental groups

Groups	Normal group	ACR group	SPI encapsulated FA	Nano SPI encapsulated FA
Parameters				
ALT (U/L)	17.00 ± 0.45 ^a^	56.00 ± 0.45^b^	30.40 ± 1.33^c^	25.00 ± 0.68^d^
AST (U/L)	35.00 ± 1.84^a^	133.00 ± 10.38^b^	80.00 ± 1.41^c^	65.20 ± 2.73^c^
ALP (U/L)	80.26 ± 2.61^a^	266.29 ± 18.36^b^	115.53 ± 0.93^c^	94.63 ± 2.04 ^ac^
γ-GT (U/L)	16.70 ± 0.54 ^a^	7.59 ± 0.38 ^b^	12.90 ± 0.37^d^	15.43 ± 0.47^a^
Total protein (g/dl)	5.45 ± 0.39 ^a^	4.58 ± 0.25 ^b^	4.85 ± 0.34 ^a^	5.02 ± 0.11 ^a^
Albumin (g/dl)	4.02 ± 0.03 ^a^	3.26 ± 0.05 ^b^	3.50 ± 0.01^d^	3.92 ± 0.10 ^a^
Total bilirubin (mg/dl)	0.25 ± 0.03 ^a^	0.83 ± 0.07 ^b^	0.54 ± 0.06 ^c^	0.41 ± 0.03 ^c^
Direct bilirubin (mg/dl)	0.04 ± 0.02 ^a^	0.27 ± 0.02 ^b^	0.18 ± 0.019 ^c^	0.14 ± 0.01 ^c^

In each row, the use of the same letters indicates a non-significant difference, while different letters signify a significant difference at *p* < 0.05. The data is presented as mean values ± standard error for each group, with six rats in each group

Table 3 shows that, the ACR group exhibited significant (*p* < 0.05) disruptions in liver function, with ALT, AST, ALP, and total bilirubin increasing by 3.29-, 3.8-, 3.32-, and 3.32-fold, respectively, compared to the normal group. serum γ-GT was significantly reduced by 54.6%, and albumin decreased by 19%, reflecting hepatic damage. Treatment with **SPI** encapsulated FA significantly reduced serum ALT (45.7%), AST (39.8%), ALP (56.6%), and total bilirubin (34.9%) while partially restoring albumin (7.4%) and γ-GT (70.0%) compared to the ACR group. On the other hand, **Nano SPI** encapsulated FA demonstrated greater amelioration, reducing ALT, AST, ALP, and total bilirubin by 55.4%, 51%, 64.5%, and 50.6%, respectively, while restoring albumin and γ-GT to near-normal levels. These findings underscore the enhanced hepatoprotective effects of nano-encapsulation in mitigating ACR-induced toxicity.

**Table 4:** Hepatic oxidative, antioxidant, and inflammatory mediators of different experimental groups

Groups	Normal group	ACR group	SPI encapsulated FA	Nano SPI encapsulated FA
Parameters				
MDA (nmol/g)	14.20 ± 0.66 ^a^	35.00 ± 1.41 ^b^	24.40 ± 0.68 ^c^	19.33 ± 0.67 ^d^
GSH (mg/g)	115.40 ± 1.63 ^a^	54.97 ± 1.45 ^b^	76.98 ± 0.90 ^c^	65.97 ± 1.07 ^d^
GPx (U/g)	35.13 ± 1.60 ^a^	14.29 ± 1.72 ^b^	21.59 ± 1.11 ^c^	27.02 ± 1.27 ^d^
CAT (U/g)	465.20 ± 5.00 ^a^	202.26 ± 1.94 ^b^	286.69 ± 5.28 ^c^	369.63 ± 2.91 ^d^
SOD (U/g)	22.50 ± 1.58 ^a^	10.46 ± 0.19 ^b^	14.05 ± 0.34 ^c^	18.47 ± 0.37 ^d^
NO (nmol/g)	61.20 ± 2.97 ^a^	144.77 ± 2.38 ^b^	34.01 ± 2.13 ^c^	28.39 ± 2.31 ^d^
IL-6 (ng/g)	12.88 ± 0.29 ^a^	32.42 ± 0.20 ^b^	27.63 ± 0.14 ^c^	20.54 ± 0.19 ^d^
TNF-α (ng/g)	14.12 ± 0.12 ^a^	36.77 ± 0.15 ^b^	30.82 ± 0.14 ^c^	23.11 ± 0.13 ^d^

In each row, the use of the same letters indicates a non-significant difference, while different letters signify a significant difference at *p* < 0.05. The data is presented as mean values ± standard error for each group, with six rats in each group

The ACR group demonstrated significant (*p* < 0.05) increases in hepatic oxidative stress and inflammatory markers, with MDA levels rising 2.46-fold, NO increasing 2.37-fold, IL-6 elevating 2.52-fold, and TNF-α increasing 2.60-fold compared to the normal group. Antioxidant parameters, including GSH, GPx, catalase, and SOD, were significantly reduced by 52.4%, 59.3%, 56.5%, and 53.5%, respectively, indicating compromised antioxidant defense. Treatment with **SPI** encapsulated FA significantly mitigated these effects, reducing MDA by 30.3%, NO by 76.5%, IL-6 by 14.8%, and TNF-α by 16.2%, while enhancing GSH, GPx, CAT, and SOD by 40.0%, 51.1%, 41.7%, and 34.3%, respectively, compared to the ACR group. **Nano SPI** encapsulated FA showed superior ameliorative effects, further reducing hepatic MDA by 44.8%, NO by 80.4%, IL-6 by 36.6%, and TNF-α by 37.15.0%, with greater enhancements in GPx (89.1%), CAT (82.7%), and SOD (76.5%) Table 4. These findings highlight the potent antioxidant and anti-inflammatory properties of nano-encapsulation.

### DNA fragmentation in hepatic tissue

**Fig. 5:**
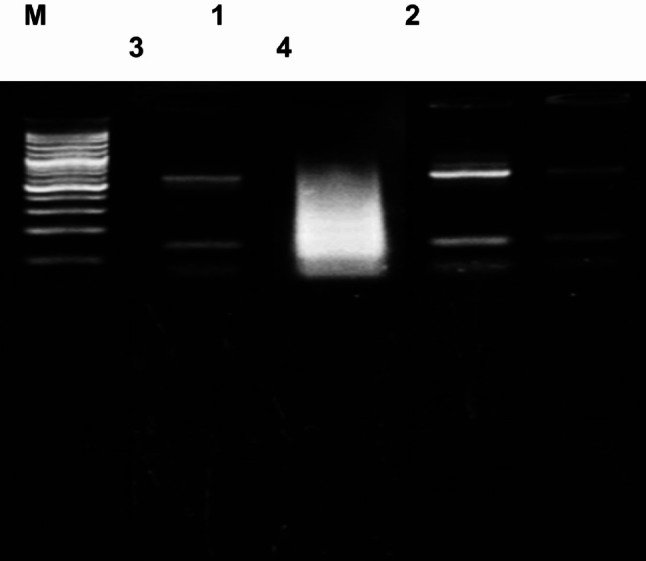
Agarose gel electrophoresis showed migration and DNA fragmentation patterns for isolated DNA from hepatocytes of all studied groups. M: DNA ladder, Lane 1: Control group DNA, Lane 2: ACR group DNA, Lane 3: SPI encapsulated FA group DNA, Lane 4: Nano-SPI encapsulated FA group DNA

Hepatic DNA fragmentation was tested by agarose gel electrophoresis. The results in Fig. 5; Table 5 revealed a highly significant (*p* < 0.0001) intra-nucleosomal DNA fragmentation in hepatocytes of the ACR group, evidenced by high smear in the DNA band and low OD value at 260 nm, compared to the control and treated groups. In contrast, both treated groups revealed intact DNA, with a minor smear observed in the SPI-FA-treated group. Surprisingly, the sesame nano-encapsulated form of FA recorded a strong protective effect against DNA damage compared to the control group, evidenced by a sharp, clear DNA band and higher OD at 260 nm. The results indicated that nano-encapsulation enhances the protective effect of the sesame protein of FA against ACR-induced genotoxicity.

### Comet assay analysis

**Fig. 6:**
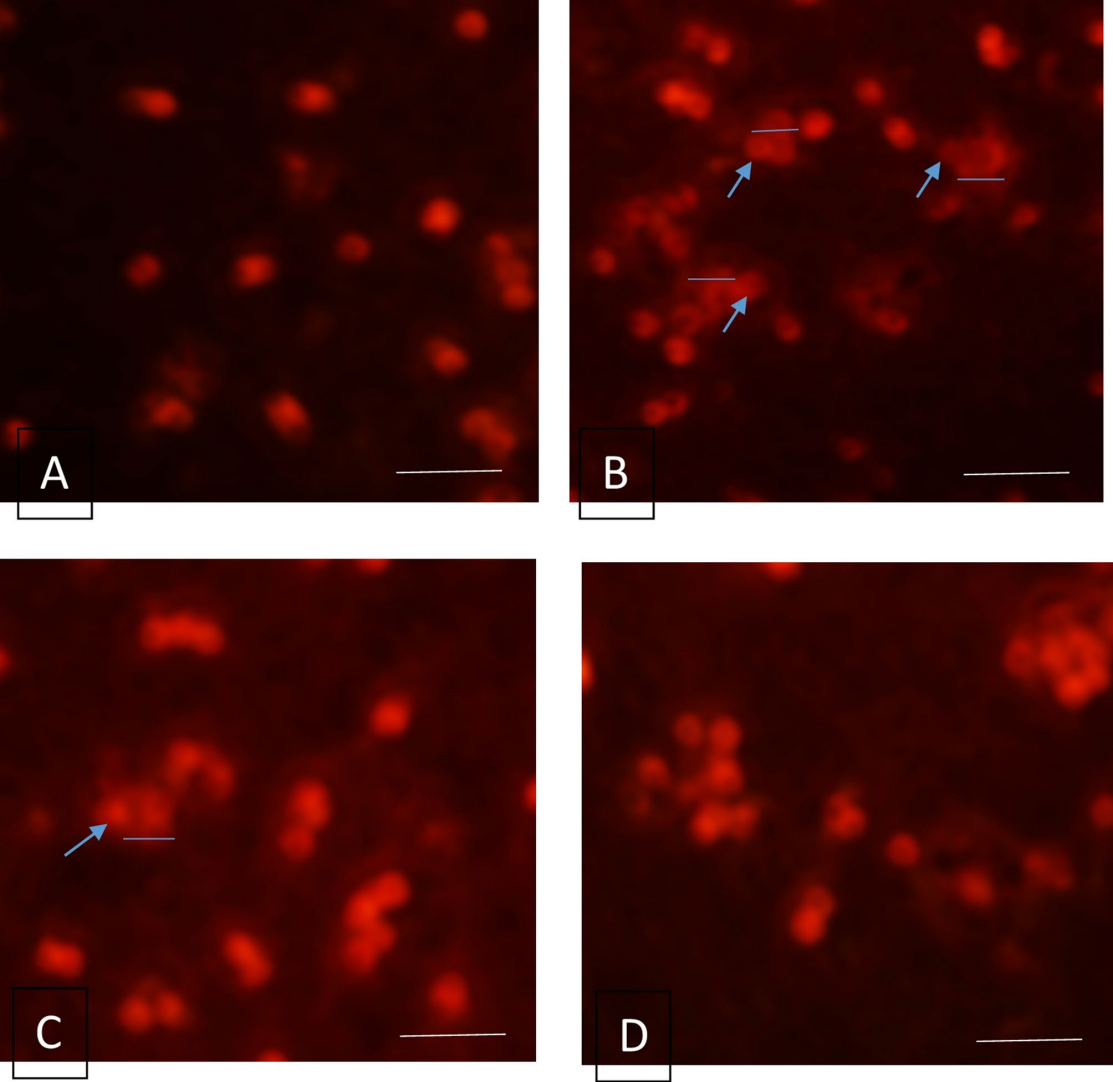
Fluorescence photomicrograph of hepatocytes for all the studied groups after single-cell gel electrophoresis (comet assay) process. (**A**) control group (intact DNA), (**B**): ACR group (Degenerated DNA; DNA head at the blue arrow and DNA tail at a blue straight line), (**C**): **SPI** encapsulated FA-treated group (Degenerated DNA; DNA head at the blue arrow and DNA tail at blue straight line), (**D**): Nano-**SPI** encapsulated ferulic acid- treated group (intact DNA), Scale bar 50 nm

DNA damage was also detected in rat hepatocytes using an alkaline comet assay. Figure 6 (A, B, C, D) and Table 5 showed a marked increase (*p* < 0.0001) in Tail length and Tail% (% DNA in tail) among ACR-treated rats, compared to all experimental groups. Treatment with SPI encapsulated FA and nano-SPI encapsulated FA significantly (*p* < 0.0001) mitigated the genotoxic effect of ACR on hepatocyte DNA by reducing the % DNA in the comet tail length, with % changes reached 56.20% and 57.8, respectively, compared to the ACR group. Nano-SPI-encapsulated FA recorded a superior effect than Sesame protein-encapsulated FA in DNA protection against DNA damage caused by acrylamide exposure.

**Table 5:** OD260 and comet parameters: tail length (µm), tailed %, and untailed% for DNA samples in all experimental groups

Groups	OD260 nm Mean ± SE	Tail length (µm)	Tailed %	Untailed%
Control	1.57 ± 0.22^a^	1.78^d^	2.8^d^	97.2^a^
ACR	0.40 ± 0.05^c^	13.4^a^	21.5^a^	78.5^d^
SPI-FA	1.11 ± 0.11^b^	5.87^b^	6.5^b^	93.5^c^
NSPI-FA	1.85 ± 0.046 ^a^	4.65^c^	4.3^c^	95.7^b^

SPI-FA: Sesame protein encapsulated ferulic acid; NSPI-FA: Sesame protein Nano-encapsulated ferulic acid

The same superscript letters mean non-significant

Least significant difference (LSD) at *p* < 0.05

### Histopathological results

**Fig. 7:**
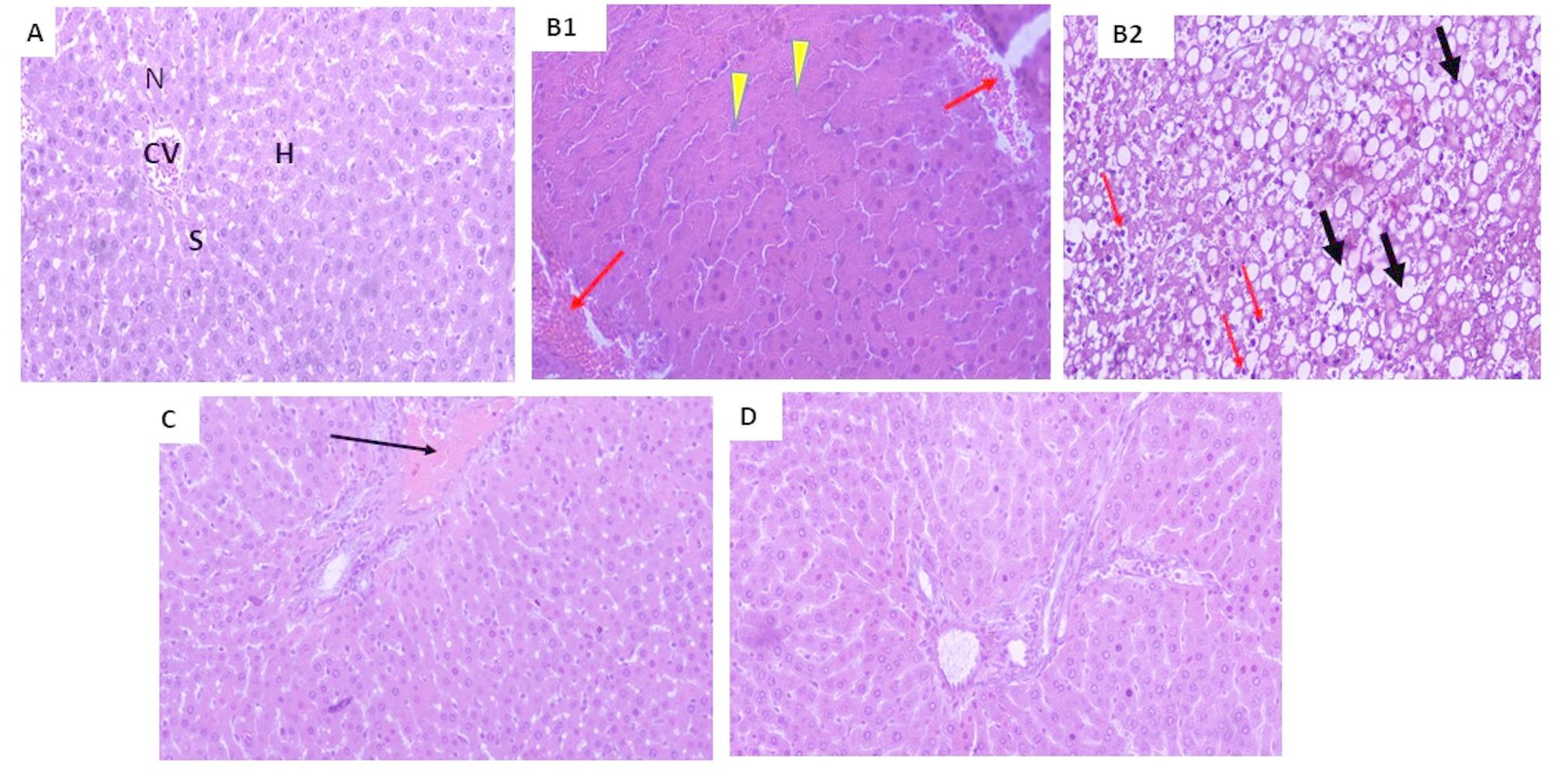
Photomicrographs of liver slices from (**A**) control rats reveal normal hepatic tissue histological structure in the form of normal hepatocyte strands (H), nuclei (N), sinusoids (S) and central vein (CV); **B1** rats that received acrylamide displaying degenerative alterations and necrotic changes of hepatocytes (yellow arrow heads), and hemorrhage (red arrow), **B2** Steatohepatitis of ARC treated rat hepatocytes with fatty degeneration, steatosis up to cell ballooning (black arrow) and scattered inflammatory cell infiltrate with Kupffer cell hyperplasia (200x) (red arrow), rats pre-treated with SPI encapsulated FA group (**C**) showing improvement of histopathological picture with slight portal congestion (black arrow) and nano-SPI encapsulated FA group (**D**) showing dramatic improvement with no histological alterations. (Scale bar 50 μm)

The results of histopathological examination are shown in Fig 7 (A, B1, B2, C, D). There were no structural alterations found in the liver of the control group. Normal hepatic cells with preserved cytoplasm, a large nucleus, and a central vein were present in the liver tissue.

In comparison to the control group, all of the tissue sections taken from the liver of rats given ACR displayed abnormal liver architecture, central vein congestion, fatty degeneration, inflammatory cellular infiltration in the portal area and between hepatocytes, sinusoidal dilatation and congestion, and Kupffer cell hyperplasia.

Additionally, compared to the ACR -treated group, the SPI encapsulated FA group exhibited a reversal of hepatic abnormalities, primarily vacuolar degeneration. Nano -SPI encapsulated FA group exhibited more improvement compared to SPI encapsulated FA form group, hepatic tissue was comparable to control group.

**Fig. 8:**
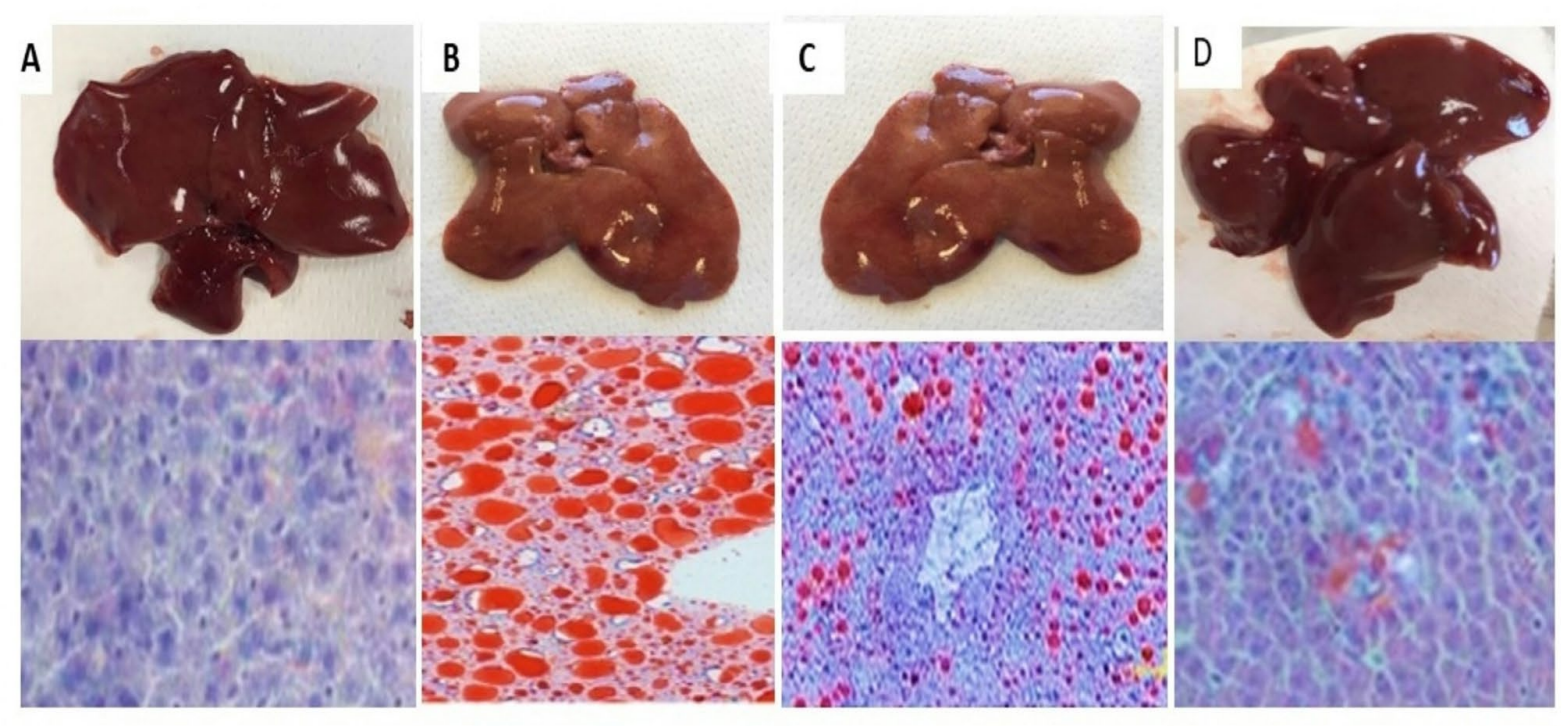
Upper panel showing a gross picture of liver from different groups. The lower panel illustrates Oil red O staining of the liver in different experimental groups: (**A**) control group, (**B**) ACR treated group showing increased fatty tissue as appeared in gross picture by increase liver brightness and marked oil red oil stain, (**C**) SPI encapsulated FA group showing less staining, (**D**) Nano -SPI encapsulated FA group with great improvement and minimal stain

The gross view of treated liver from different studied groups is displayed in Fig. 8(A, B, C, D). Additionally, it displays the findings of histological analyses that use oil red O staining to identify fat deposits. Compared to the control group, the liver of the ARC-treated rats had a greater degree of lipid accumulation that even appears in the gross microscopic picture of liver tissue as a bright greasy enlarged liver. In contrast to the acrylamide group, the SPI encapsulated FA group evidently demonstrated a significant reduction in hepatosteatosis. In contrast to the ARC and ferulic encapsulated groups, the nano -SPI encapsulated FA group showed greater improvement and nearly full recovery from hepatosteatosis.

**Fig. 9:**
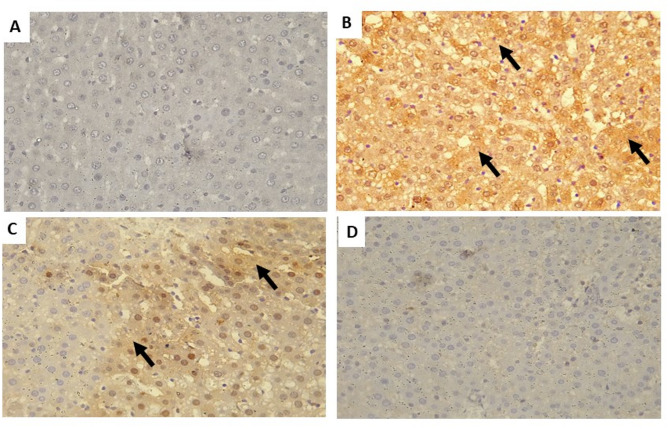
Immunohistochemical staining of the apoptotic marker Caspase − 3 in liver sections from experimental groups (x400), appeared as brown cytoplasmic immunoreactions and counterstained with Hematoxylin.; Control group (**A**) showing few scanty immunoexpression of Caspase − 3 (arrows). ACR treated group (**B**) showing strong positive immunoexpression of Caspase − 3 (arrows) within the liver cells indicating increased cell apoptosis. SPI encapsulated FA group (**C**) showing a few scanty immunoexpression of Caspase − 3 within the hepatic cells. nano -SPI encapsulated FA group (**D**) showing more decrease in the expression of Caspase − 3 immunoexpression within most hepatic cells Scale bars = 50 μm

In reference to the immunohistochemistry analysis and apoptosis assessment, ARC exhibits a robust positive reactivity to caspase-3, compared to the control group. Comparing the SPI encapsulated FA group to the ARC group, the former showed lower caspase immunoreactivity. In contrast to the SPI encapsulated FA group, nano -SPI encapsulated FA -treated hepatic tissues demonstrated no immunoreactivity as seen in Fig. 9 (A, B,C, D).

## Discussion

Sesame protein isolate (SPI), prepared from defatted sesame flour, is utilized for human and animal nutrition due to its high-quality protein content [20]. SPI contains about 89.4 ± 0.36% proteins as SPI (88.98 ± 0.13%) recorded by Mustafa & Hüseyin [53] who reported 88.98 ± 0.13% protein content. The formation of nano-SPI is due to the utilization of ultrasonic waves, which produce high-temperature, high-pressure, and high-frequency vibrations. These vibrations rapidly change the structural and functional properties of proteins through mechanical and cavitation effects. Ultrasonic waves can also disrupt peptide bonds, induce subunit dissociation or aggregation, and obstruct noncovalent interactions between natural protein molecules [21].

The spectrum of SPI was composed of several bands located at 3273, 2962 cm⁻¹ which are assigned to the stretching vibration of OH groups, C-H groups. Bands at 1669, 1632, 1537 cm⁻¹ correspond to peptide group segments (the amide I band C = O stretching, and amide II bands C-N stretching and N-H stretching) respectively [54, 55]. Similar bands have been observed in other types of proteins as whey protein isolates (WPI) [56]. The most sensitive spectral region is the amide I band (1700–1600 cm⁻¹), which is sensitive to protein secondary structural components [57]. The spectrum of SPI nano form showed a similar pattern of bands as those of SPI, but with a change in band intensity. This could be due to the exposure of the protein to ultrasonic waves, which formed nanoparticles with more functional groups than SPI. The FTIR spectrum of both encapsulated forms shows some frequency and intensity differences, suggesting some alterations in the amino acid contributions and protein conformation due to addition of FA and sodium alginate.

SEM and POM confirmed that the ultrasonic waves applied to SPI successfully prepared the SPI nanoparticles. Particles between 1 and 1000 nm in size are known as polymeric nanoparticles (NPs), and they can have active substances either surface-adsorbed onto or trapped inside the polymeric core [58].

Encapsulation efficiency (EE) of nano SPI encapsulated was higher than that of SPI encapsulated, illustrating the importance of nano form particles. Antioxidants for the soluble capsules also showed that the scavenging activity of DPPH and ABTS percentage was higher in SPI encapsulated than in nano-SPI encapsulated form, which correlates with the encapsulation efficiency. This suggests that the release of FA is faster and higher in SPI capsules than nano SPI-capsules. Encapsulation allows for additional coating or surface alterations of the biomaterial in addition to enclosing the agent in its inner structure to protect the encapsulated materials [59].

This study investigated the hepatoprotective effects of FA encapsulated with sesame protein, both in its capsulated and nano forms, in combating ACR-induced liver toxicity. ACR, a widely recognized hydrophilic toxicant present in various foods, is known for its ease of tissue permeability upon ingestion, with the liver particularly vulnerable due to its central role in metabolism [60]. Our results indicate that both FA formulations encapsulated with sesame protein successfully reduce ACR-induced hepatic damage in rats.

ACR exposure compromises antioxidant defenses by promoting reactive oxygen species (ROS) generation, leading to oxidative stress and lipid peroxidation, contributing to cellular damage [61]. Consistent with this, oral administration of ACR in our study significantly elevated serum liver enzymes (AST, ALT, γ-GT, ALP), total and direct bilirubin, MDA, and NO while decreasing antioxidant markers such as GSH, GPx, catalase, and SOD. These findings align with previous studies demonstrating ACR’s role in ROS induction and oxidative stress [62, 63]. The increase in ROS also leads to cell membrane disruption, as evidenced by lipid peroxidation and MDA formation, which exacerbate inflammatory responses [64].

ACR-induced oxidative stress triggers inflammatory pathways, resulting in elevated levels of inflammatory cytokines TNF-α and IL-6, likely due to NF-κB pathway activation—a process supported by prior studies on oxidative stress and inflammation [65, 66]. Similarly, Nan et al. [67] found that ACR-induced liver inflammation elevated TNF-α and IL-1β levels via NF-κB signaling. However, the administration of FA encapsulated with sesame protein, particularly in nano form, significantly decreased these inflammatory cytokines, indicating anti-inflammatory effects.

Both FA formulations improved oxidative stress markers and liver function. Notably, the nano form exhibited enhanced efficacy, significantly raising antioxidant enzyme levels (SOD, GSH-Px, CAT) while lowering MDA and NO levels. This supports previous research showing FA’s antioxidant and anti-inflammatory properties via Nrf2 upregulation and NF-κB downregulation, promoting a balance between oxidative and inflammatory pathways [68]. The phenolic structure of FA further facilitates its free radical scavenging capabilities, enhancing its effectiveness against oxidative stress and liver damage [69].

The role of sesame protein as an encapsulation medium also contributed to the observed hepatoprotective effects. Sesame protein, rich in sulfur-containing amino acids such as methionine, supports antioxidant defenses by potentially preserving antioxidant enzyme activity [70, 71]. A study by Yang et al. [72] showed that sesame protein hydrolysates have antioxidative and anti-inflammatory properties, which likely potentiated FA’s effects on liver enzyme and bilirubin levels in this study. These findings are further corroborated by a study from Wang [73], which illustrates FA derivatives’ ability to restore liver function, lessen liver injury, and alleviate fibrosis. Importantly, our study showed that nanoencapsulation further enhanced FA’s hepatoprotective effects, likely due to improved bioavailability and efficacy, consistent with a previous report on the biocompatibility and efficiency of lipid-based nanoparticles [71].

Histopathological findings in this study proved that liver exposure to ACR induces hepatic toxicity in the form of steatosis and fatty degeneration up to cell ballooning which was in agreement with Liu et al. [74], who reported that exposure to ACR could induce oxidative stress to the liver and disrupt the metabolism of lipids (including cholesterol metabolism, sphingolipid metabolism, glycerophospholipid metabolism and fatty acid β-oxidation metabolism). Other study revealed that ACR induces hepatic tissue alterations that were represented by light microscopy as fatty deposits, congested central vein and cell vacuolization [75].

The herein work claimed that ACR induces hepatic structure deterioration, the microscopical degenerations that show liver damage, such as the increased necrosis, inflammation and hemorrhagic areas were more common and apparent when compared to other groups which was consistent with Donmez et al. [76]. Moreover, our results were in agreement with Abdulal et al. [77] who reported that histopathological liver tissue examination showed that FA ameliorated the appearance of vacuolated cytoplasm, reduced apoptotic nuclei, and necrotic nodules in rat liver. Also, Junhui Yuan et al. found that FA reduced the degree of necrosis and bleeding and alleviated the pathological changes caused by acetaminophen in rat liver [78]. Previous study showed that SPI decreases membrane lipid peroxidation in rats [79]. Also, Li et al. reported that feeding rats with black and white sesame caused recovery from fatty liver which may be partially agree with our study [80].

Inflammation and oxidative stress are crucial for the pathological development of hepatic steatosis, which can lead to cirrhosis, non-alcoholic fatty liver disease, or non-alcoholic steatohepatitis [81]. Sesame derivatives such as SPI also have anti-inflammatory and ameliorate oxidative stress consequently improving liver functions and histopathological picture as well [82].

Caspases, also referred to as cysteine proteases, are members of the interleukin-1β-converting enzyme family and are crucial for inducing apoptosis. The mammalian caspase family comprises 14 members, referred to as caspase-1-14. While some caspases are essential for apoptosis, others are not. The majority of caspases are involved in inflammation, differentiation, proliferation, or cell survival [83]. Either an intrinsic or an extrinsic pathway can trigger apoptosis in animals. Each of these routes activates the downstream effector caspase-3. It is essential to the cell apoptosis execution phase [84]. Oxidative stress may activate caspases, such as caspase-3, which would promote cell death [85].

Yaun et al. results are in line with the present work as Caspase-3 activity detection showed that FA pretreatment decreased the degree of apoptosis significantly in acetaminophen-induced hepatotoxicity in mice [86]. The present results are in accordance with Luo et al. [87] who reported that FA ameliorate diabetes induced hepatosteatosis in rats.

Additionally, by lowering hydroxyl radicals, peroxynitrite, superoxide anion, and nitric oxide, SPI reduced the production of free radicals. In hepatic damage, it preserved GSH and reduced lipid peroxidation [88]. Proteins and peptides derived from sesame seeds have been reported for medicinal properties, including antioxidant [89]. According to Adebisi et al., SPI provides major pharmacological advantages and health benefits for the entire body, particularly the liver [90]. The same author stated that Sesame proteins contain anti-inflammatory, anti-apoptotic and antioxidant ingredients that may support our findings.

Our findings uncover the genotoxic effects of ACR on hepatic DNA and the potential protective role of SPI-encapsulated FA and its nano-encapsulated form. The results revealed significant DNA fragmentation and damage in ACR-exposed hepatocytes, as evidenced by both the agarose gel electrophoresis and the comet assay. Treatment with SPI-encapsulated FA and nanoform significantly mitigated this damage, with nano-encapsulated form exhibiting superior protection. These findings align with the study of Eisenbrand [91] on ACR-induced genotoxicity and the protective effects of antioxidants. The DNA fragmentation observed in the ACR group supports previous studies indicating that ACR and its metabolite glycidamide can induce significant DNA damage. According to Tareke et al. [92], glycidamide forms DNA adducts contributing to mutations and carcinogenesis. Moreover, several studies reported DNA strand breaks and oxidative stress in hepatocytes following ACR exposure [93, 94] aligned with the significant smear pattern observed in the DNA bands and increased tail length in comet assay in the present study. FA is known for its antioxidant and free radical scavenging properties [95].

Previous studies by Ou and Kwok [96] demonstrated that FA reduces oxidative damage by neutralizing ROS and protecting cellular macromolecules. However, its limited bioavailability restricts its efficacy. In this study, the encapsulation of FA within sesame protein significantly enhanced its protective effect, as reflected in the reduced DNA fragmentation and comet assay parameters. These results are consistent with Garav and his colleagues [97] revealed that encapsulating phenolic compounds enhances their stability, bioavailability, and antioxidant capacity, which aligns with our findings. The superior protective effect of nano-encapsulated form compared to encapsulated form can be attributed to the enhanced cellular uptake and prolonged antioxidant activity provided by the nano-encapsulation process. Nano-encapsulation improves the solubility and bioavailability of FA, allowing for more efficient delivery and sustained protection against oxidative stress and DNA damage. Our results are in harmony with the study of Zhang et al. [98] who reported that nano-carrier systems significantly improve the therapeutic efficacy of antioxidants in protecting DNA integrity.

The protective effects of both encapsulated forms can be explained by their ability to reduce oxidative stress and prevent the formation of DNA adducts. The antioxidant activity of FA likely counteracts the ROS generated by ACR metabolism, thereby preventing oxidative damage to DNA. Additionally, sesame protein may contribute to this effect through its own antioxidant properties, as suggested by Kim et al. [99].

## Conclusion

This study suggests the potential of using FA encapsulated within SPI, particularly in its nano form, for a protective effect against ACR-induced liver toxicity and genotoxicity. By targeting the oxidative stress induced by ACR, the research indicates potential hepatoprotective and genoprotective effects of this nutraceutical in this model. The nanoencapsulation appears to enhance the bioavailability and antioxidant activity of FA, potentially offering a promising intervention for mitigating liver injuries caused by environmental and dietary ACR exposure.

Study limitation: The use of a rat model may not fully translate to human responses, and the acute duration restricts the understanding of chronic effects of acrylamide exposure. Notably, the study did not explore the impact of varying dosages of the encapsulated FA, which is crucial for determining optimal therapeutic levels. Further research, including dose-response studies, human trials, and detailed mechanistic and pharmacokinetic analyses, is necessary to validate these findings for clinical translation.

## Electronic supplementary material

Below is the link to the electronic supplementary material.

Supplementary Material 1

## Data Availability

No datasets were generated or analysed during the current study.

## Declarations

### Confirmation that all authors have approved the manuscript for submission

All authors have approved the manuscript for submission.
