# Supplementary figures and images for: Nano-encapsulated ferulic acid in sesame protein isolate alleviates acrylamide-induced liver toxicity and genotoxicity in rats via oxidative stress and DNA damage modulation
Source: BMC Pharmacol Toxicol. 2025 Jun 13;26:120. doi: 10.1186/s40360-025-00946-8 (PMC12166574; doi:10.1186/s40360-025-00946-8)

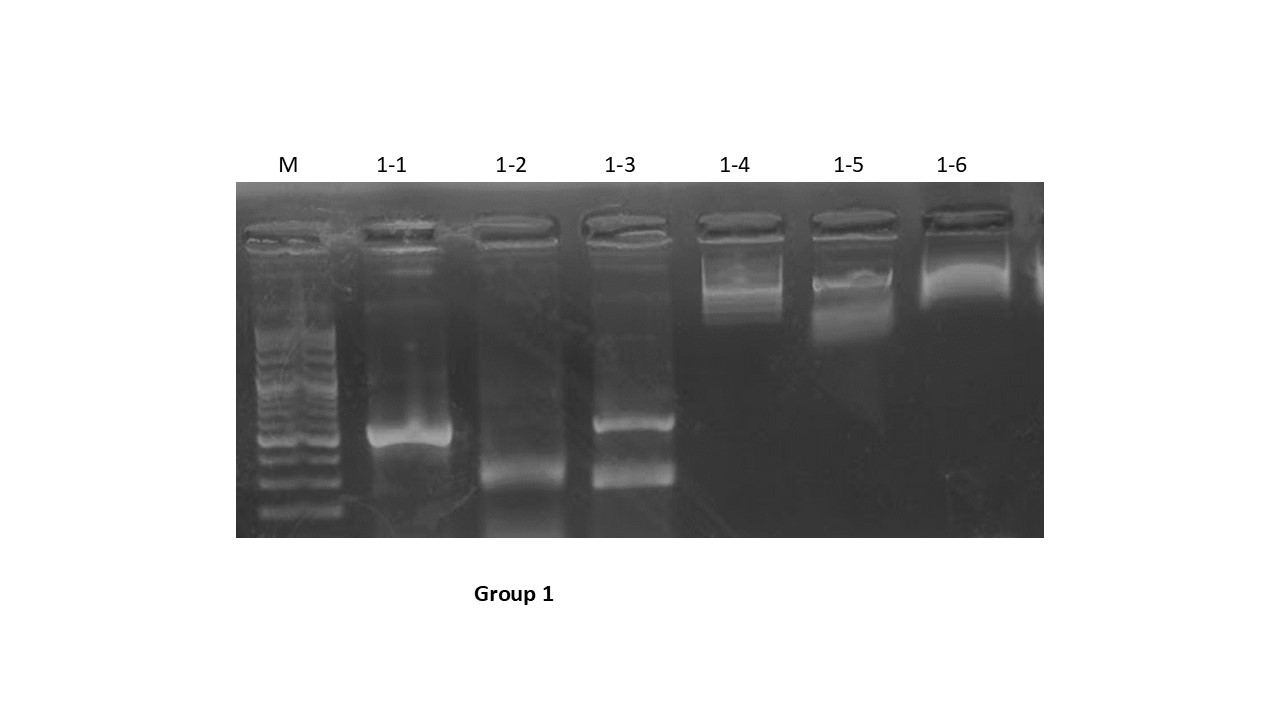


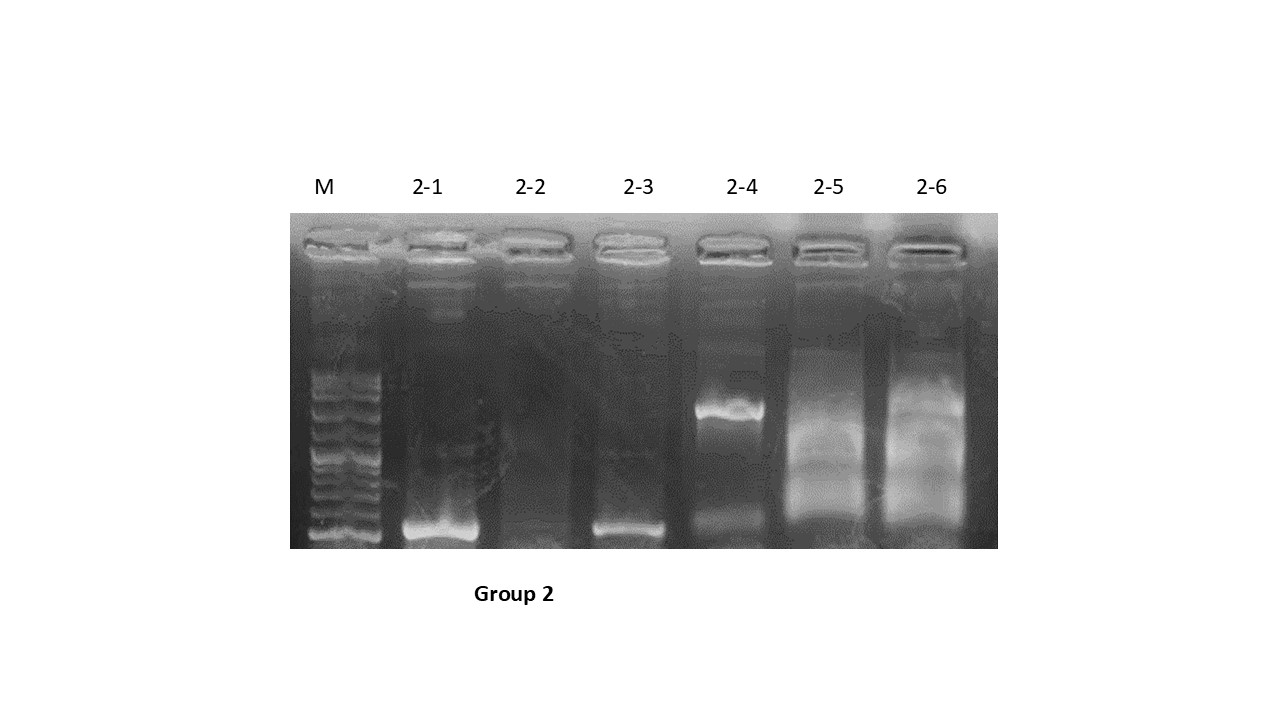


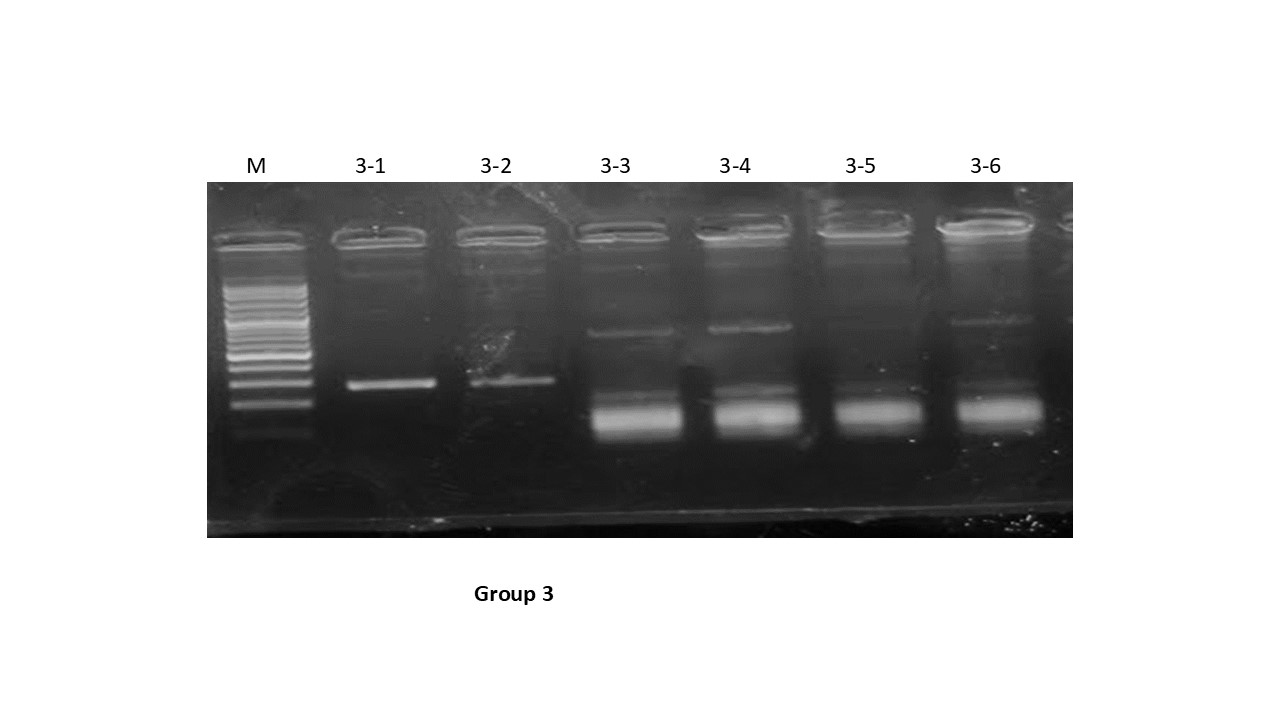


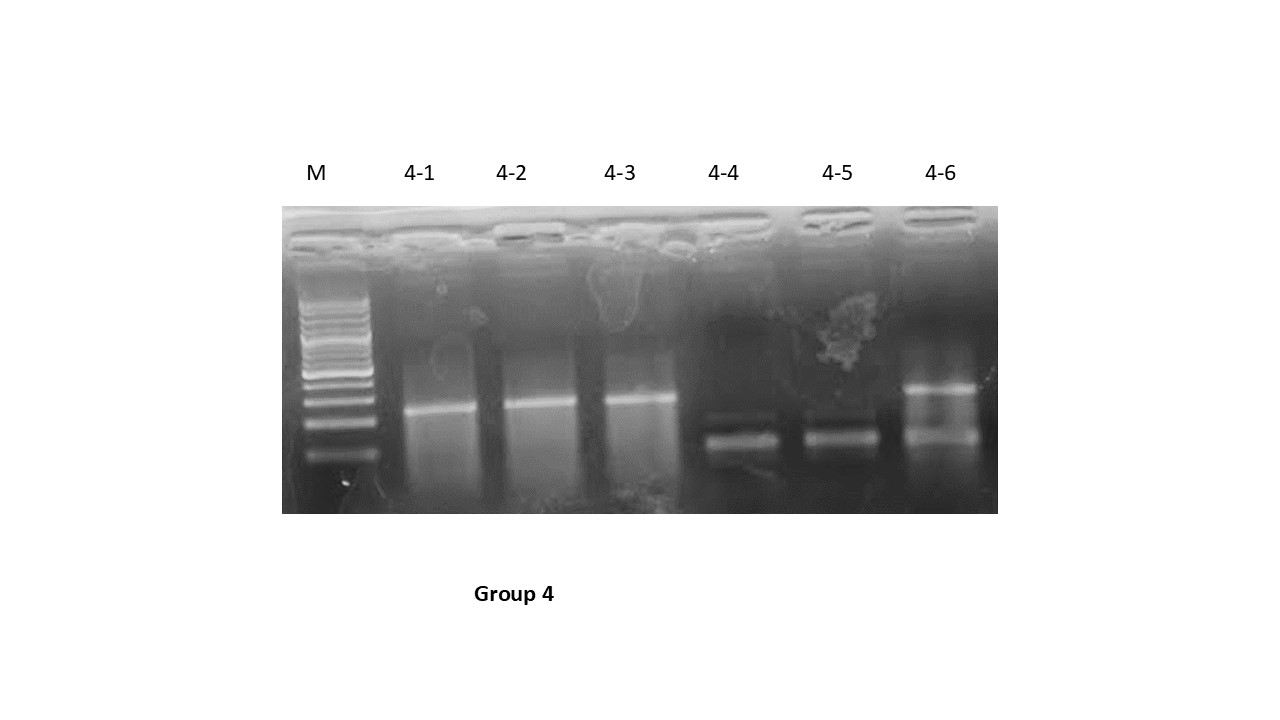


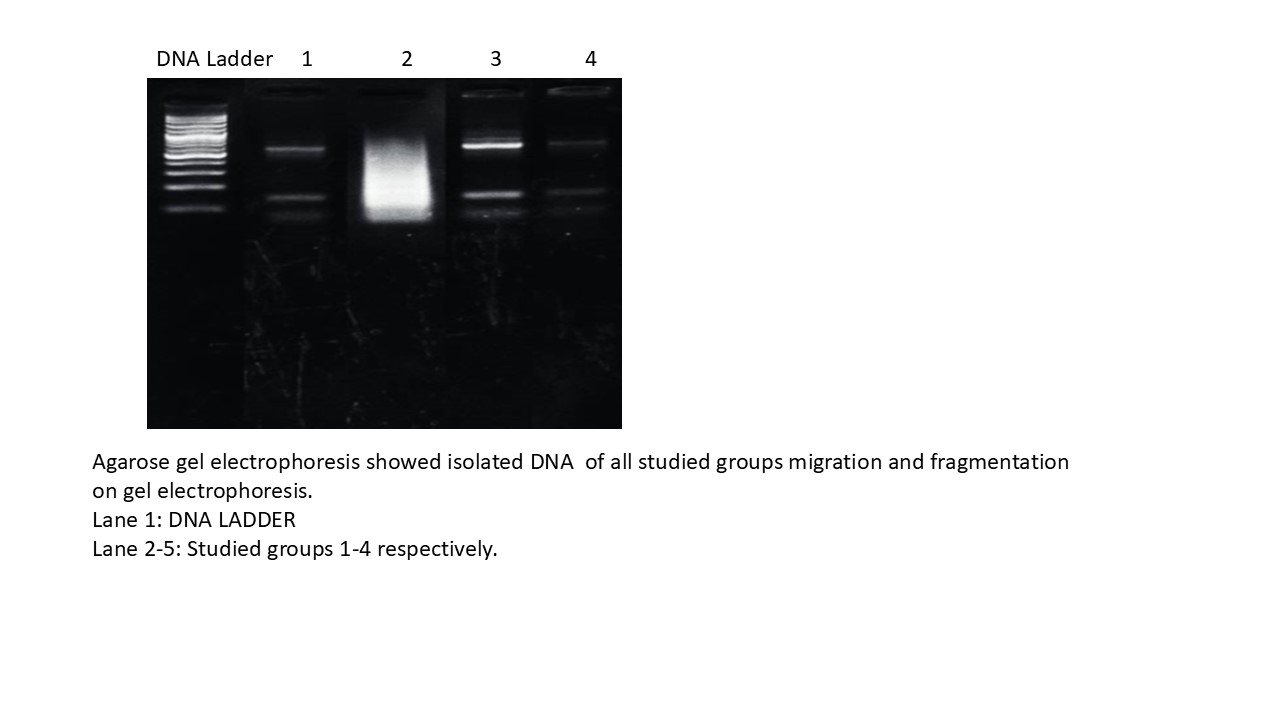


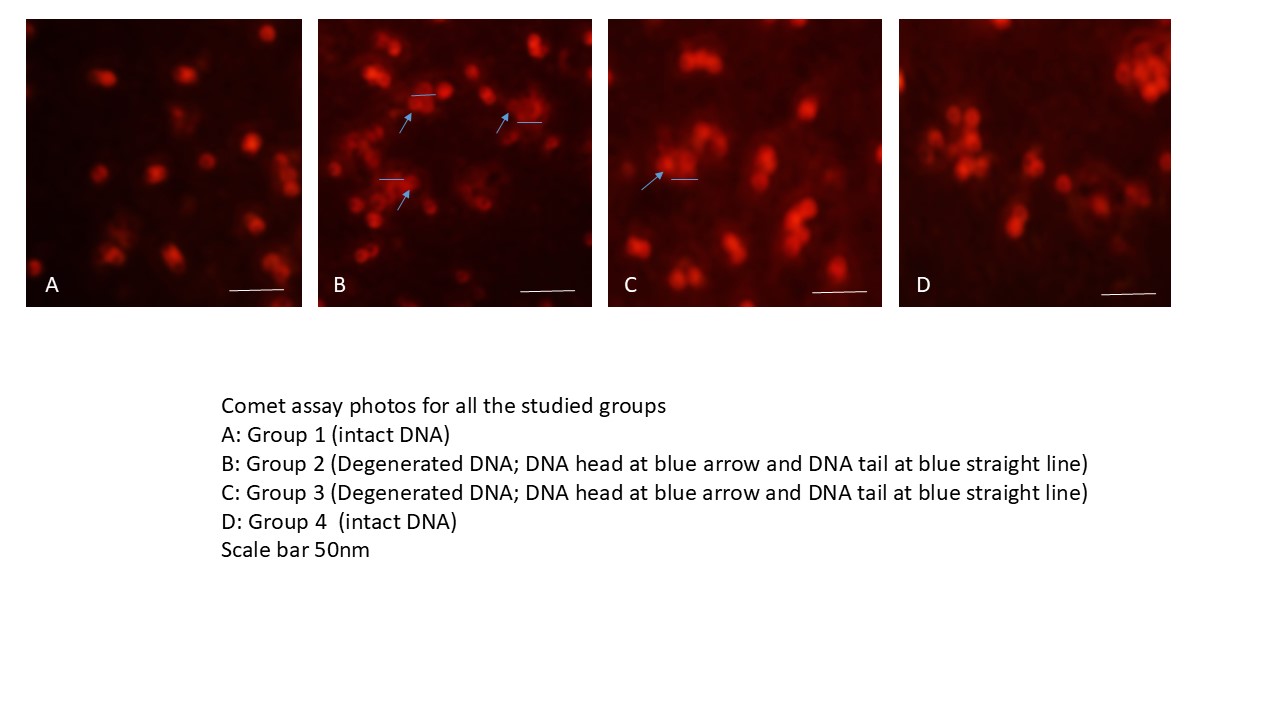

Supplement: Supplementary file 1 — Supplementary Material 1 [file 40360_2025_946_MOESM1_ESM.docx]
